# Exploring the immunogenicity of an insect-specific virus vectored Zika vaccine candidate

**DOI:** 10.1038/s41598-023-47086-9

**Published:** 2023-11-15

**Authors:** Manette Tanelus, Krisangel López, Shaan Smith, John A. Muller, Danielle L. Porier, Dawn I. Auguste, William B. Stone, Sally L. Paulson, Albert J. Auguste

**Affiliations:** 1 Department of Entomology, College of Agriculture and Life Sciences, Fralin Life Science Institute, Virginia Polytechnic Institute and State University, Blacksburg, VA 24061 USA; 2 Center for Emerging, Zoonotic, and Arthropod-Borne Pathogens, Virginia Polytechnic Institute and State University, Blacksburg, VA 24061 USA

**Keywords:** Viral infection, Vaccines

## Abstract

Zika virus (ZIKV) is an important re-emerging flavivirus that presents a significant threat to human health worldwide. Despite its importance, no vaccines are approved for use in humans. Insect-specific flaviviruses (ISFVs) have recently garnered attention as an antigen presentation platform for vaccine development and diagnostic applications. Here, we further explore the safety, immunogenicity, and efficacy of a chimeric ISFV-Zika vaccine candidate, designated Aripo-Zika (ARPV/ZIKV). Our results show a near-linear relationship between increased dose and immunogenicity, with 10^11^ genome copies (i.e., 10^8^ focus forming units) being the minimum dose required for protection from ZIKV-induced morbidity and mortality in mice. Including boosters did not significantly increase the short-term efficacy of ARPV/ZIKV-vaccinated mice. We also show that weanling mice derived from ARPV/ZIKV-vaccinated dams were completely protected from ZIKV-induced morbidity and mortality upon challenge, suggesting efficient transfer of maternally-derived protective antibodies. Finally, in vitro coinfection studies of ZIKV with Aripo virus (ARPV) and ARPV/ZIKV in African green monkey kidney cells (i.e., Vero-76) showed that ARPV and ARPV/ZIKV remain incapable of replication in vertebrate cells, despite the presence of active ZIKV replication. Altogether, our data continue to support ISFV-based vaccines, and specifically the ARPV backbone is a safe, immunogenic and effective vaccine strategy for flaviviruses.

## Introduction

The genus *Flavivirus* contains arthropod-transmitted viruses that have the potential to cause significant disease, ranging from mild flu-like symptoms to severe neurological and/or hemorrhagic complications^1^. The diverse genus contains vertebrate-infectious flaviviruses (VIFs) such as Dengue virus, West Nile virus, yellow fever virus, and Japanese encephalitis virus, as well as insect-specific flaviviruses (ISFVs), and some viruses with no known vector^1,2^. Zika virus (ZIKV) was originally isolated in 1947 in Uganda^3^ and has emerged within the last decade, causing significant outbreaks worldwide with an enormous impact on public health^4^. The first report of a symptomatic outbreak of ZIKV was in 2007 on Yap Island, Micronesia^5^. Between 2013–2014, a ZIKV epidemic occurred in French Polynesia^6,7^. In 2015, a Zika outbreak was reported in Brazil and this ultimately caused explosive outbreaks throughout Latin America and the Caribbean in 2015–2016^7–9^. Following the outbreaks, ZIKV infection was found to be associated with Guillain-Barré syndrome in adults and congenital Zika syndrome in newborns^5,10^.

Despite the importance and public health impact of ZIKV, there are no FDA-approved therapeutics or vaccines available, although several ZIKV vaccines are presently in clinical trials^11,12^. Recently, flavivirus vaccines based on chimeric insect-specific viruses have shown promising results and may offer additional vaccine candidates for consideration. Insect-specific flaviviruses (ISFVs) replicate efficiently in the arthropod vector but are incapable of replication within vertebrate hosts^13–17^. Given the ISFV backbone, these chimeric vaccines are host-restricted and cannot replicate in vertebrate cells, yet, they elicit strong immune responses when administered at high doses in vertebrates^16,18,19^. We recently produced a chimeric ZIKV vaccine candidate that includes the surface proteins of ZIKV virus (prM and E proteins) substituted into the genome of an ISFV named Aripo virus (ARPV)^20,21^. ARPV is phylogenetically classified as a dual-host affiliated ISFV (i.e., an insect-specific virus that clusters together with pathogenic flaviviruses but is host-restricted for replication in insect cells only) isolated between 2007 and 2009 in Trinidad and has been shown to be immunomodulatory in vertebrate cells^20^.

Recent studies show that the Aripo-Zika (ARPV/ZIKV) vaccine candidate retains ARPV’s natural vertebrate host restriction and is exceptionally safe in murine models^20,21^. Previous studies demonstrate that a single dose of ARPV/ZIKV provided complete protection from viremia, weight loss, and mortality in immune-competent (C57BL/6J) and immune-compromised murine models (IFNαβ^−/−^)^21^. We also showed that a single dose of ARPV/ZIKV completely protects pregnant dams after ZIKV challenge and prevents in utero transmission of ZIKV to neonates^21^. However, this study did not measure antibody titers in neonates. Here, we seek to determine if maternal antibodies are transferred from ARPV/ZIKV-vaccinated dams to their offspring and evaluate the degree of protection they are afforded.

Although ARPV/ZIKV demonstrates exceptional safety and efficacy, very little is known about (1) the minimal and/or optimal dose for achieving complete protection from ZIKV-induced disease; (2) the impact of boosters on vaccine-induced immunity; or (3) the influence of VIFs during coinfection with ARPV/ZIKV in vertebrate cells. Our results show that a dose of 10^11^ genome copies (GC) is the minimum dose required for protection from ZIKV-induced morbidity and mortality in immune-competent mice. We also demonstrate that including ARPV/ZIKV boosters did not significantly increase the short-term efficacy among vaccinated mice, and show evidence of inducing sterilizing immunity during our booster studies in all ARPV/ZIKV groups in an immune-competent mouse model. Finally, we explore in vitro coinfection studies of ZIKV with ARPV and ARPV/ZIKV and showed that ARPV and ARPV/ZIKV remain incapable of replication in vertebrate cells despite the presence of active ZIKV replication, a critically important finding when considering administering this vaccine candidate to flavivirus endemic countries.

## Results

### ARPV and ARPV/ZIKV remain incapable of replication in Vero-76 cells during coinfection with ZIKV

**Figure 1:**
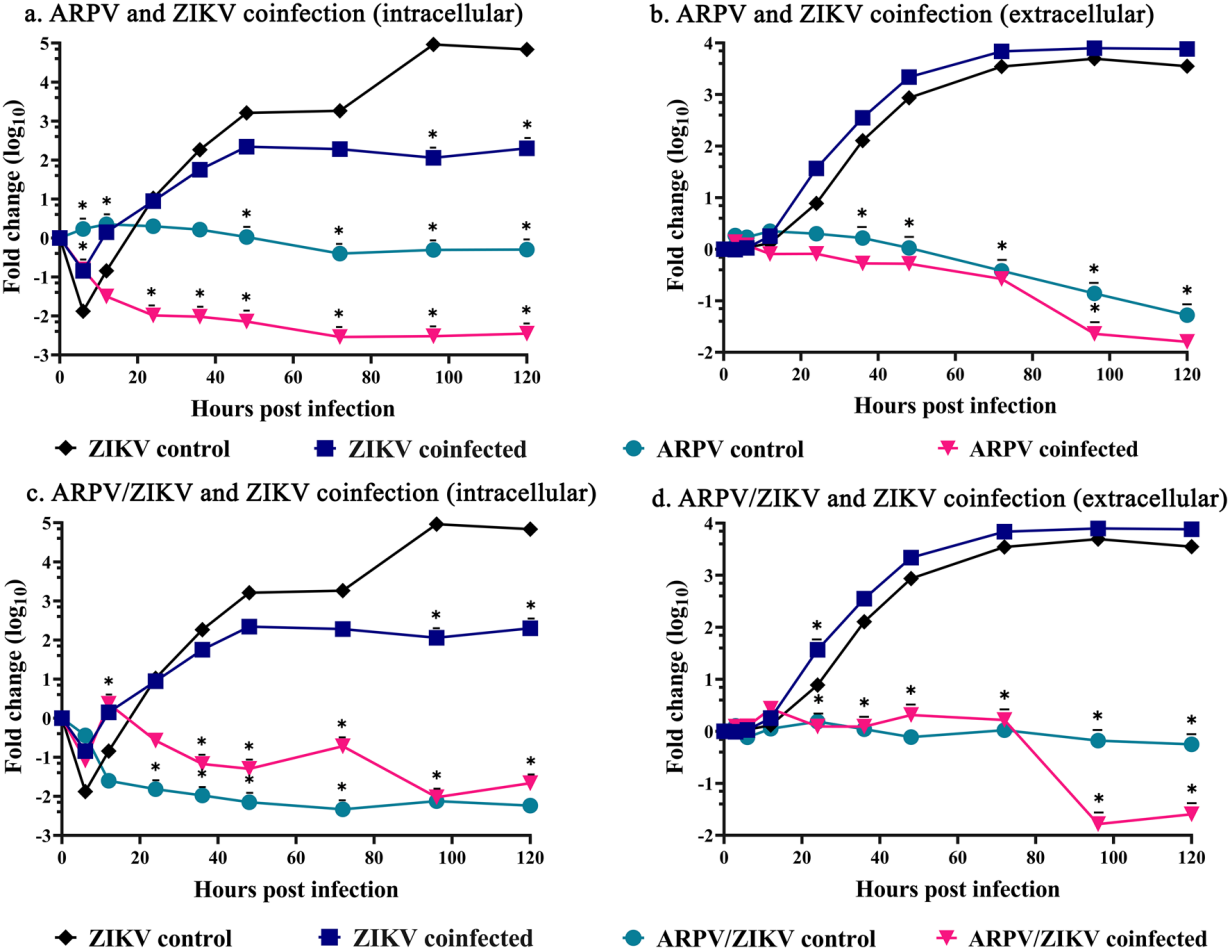
ARPV and ARPV/ZIKV do not replicate in vertebrate cells (Vero-76) during coinfection. Growth kinetics for each virus were compared for coinfected and control groups of: (**a**) ARPV and ZIKV intracellular factions, (**b**) ARPV and ZIKV extracellular factions, (**c**) ARPV/ZIKV and ZIKV intracellular factions, and (**d**) ARPV/ZIKV and ZIKV extracellular factions. Vero-76 cells were infected at a target multiplicity of infection (MOI) of 0.1 with each virus, and intracellular and supernatant fractions were taken at 0, 3, 6, 12, 24, 36, 48, 72, 96, and 120 h post-infection (hpi). Viral RNA were extracted and quantified using RT-qPCR. Data points are presented as log_10_ fold change in virus titer, where the fold change was estimated by dividing the virus titer at the indicated timepoint by the virus titer measured at the 0 h time point for that respective virus. Asterisks indicate significance compared via two-way ANOVA and mixed-effects analyses. Unless otherwise marked, asterisks indicate significance compared ZIKV control; p ≤ 0.033 (*).

Flaviviruses co-circulate in many countries, and it is not unlikely to be simultaneously exposed or coinfected with different flaviviruses^22–24^. If our vaccine were to be successful in preclinical development, it is important to determine if administering the vaccine during an active flavivirus infection could impact either virus’ replication kinetics. Therefore, to investigate the impact of ZIKV coinfection with ARPV or ARPV/ZIKV in vertebrate cells, we assessed the growth kinetics of each coinfecting virus in the intracellular and extracellular fractions of Vero-76 infected cells. ARPV and ARPV/ZIKV did not show evidence of intracellular or extracellular replication when infected alone, nor during coinfection with ZIKV (Fig. 1a–d). In contrast, ZIKV showed rapid and robust replication in both intracellular and extracellular fractions during both infection scenarios. Both intracellular and extracellular fractions showed similar growth kinetics for all viruses (Fig. 1a–d). Interestingly, ZIKV titers in the intracellular fraction was significantly lower at 96- and 120-h post-infection (hpi) during coinfection with either ARPV or ARPV/ZIKV than the control ZIKV infections (Fig. 1a, c).

### ARPV/ZIKV immunization induces protective immunity after a single dose of ≥ 10^11^ genome copies

**Figure 2:**
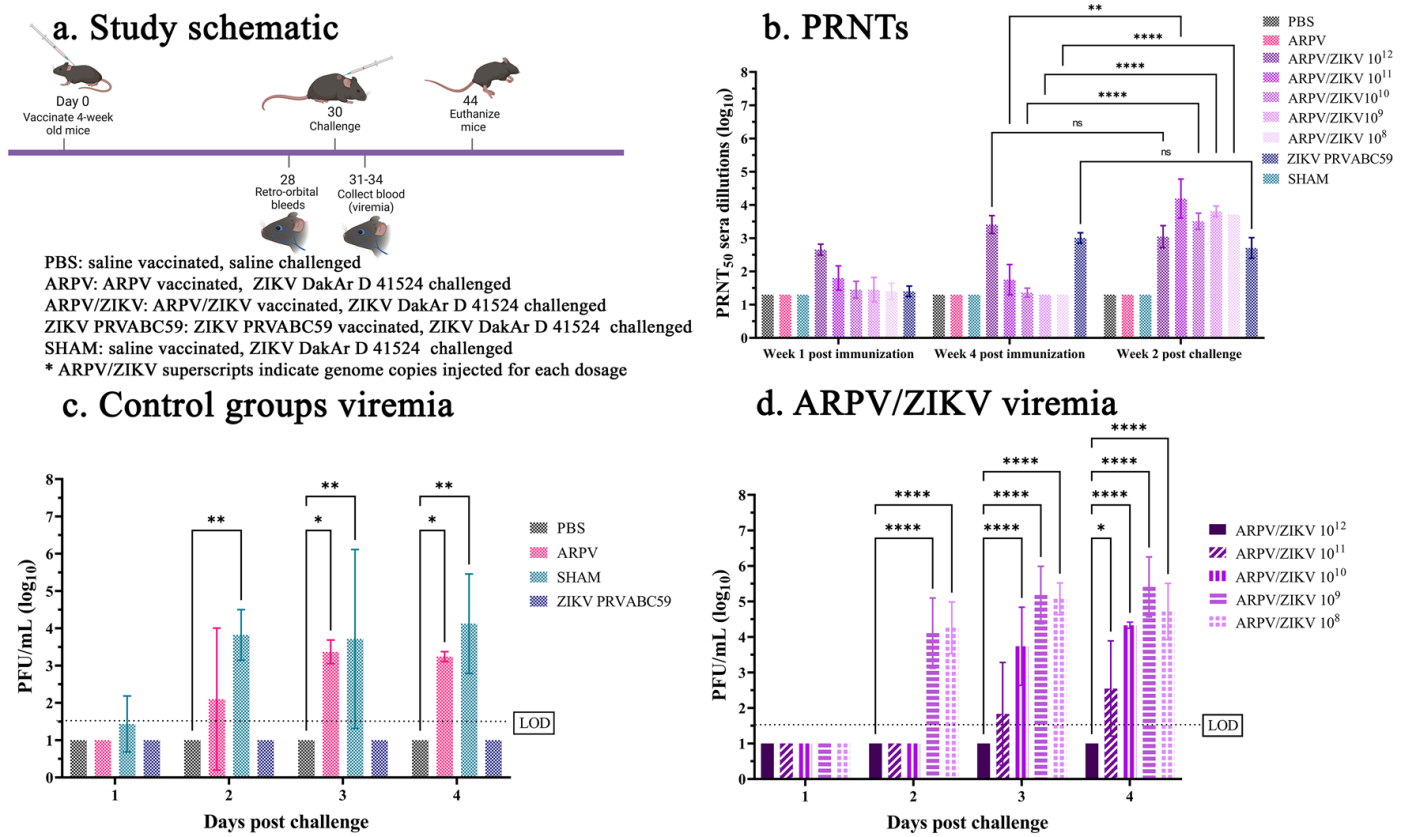
ARPV/ZIKV shows protective immunity at ≥ 10^11^ genome copies (GC). Four-week-old C57BL/6 J mice (n = 6) were inoculated subcutaneously (s.c.) with the indicated agents or with saline (PBS) as shown in study schematic (**a**). All mice except PBS were challenged with ZIKV DakAr D 41524 s.c. with 2 × 10^5^ plaque forming units (PFU) or PBS at 30 days post immunization and monitored for 14 days post challenge for weight change and survival. Sera was collected at 1 week post immunization, and 2 weeks post immunization, 4 weeks post challenge respectively and ZIKV-specific Plaque Reduction Neutralizing Tests (PRNT_50_) were done (**b**). Viremia was measured from sera collected on days 1–4 post challenge (**c**,**d**). Limit of detection is indicated by the dotted line. Data points represent mean values and error bars represent the standard deviation. Significance was determined by two-way ANOVA and mixed-effect analyses. Unless otherwise marked, asterisks indicate significance compared to healthy controls (PBS): not significant (ns), p ≤ 0.033 (*), p ≤ 0.002 (**), p ≤ 0.0002 (***) and p ≤ 0.0001 (****). Unless otherwise marked, there was no significant difference between PBS and ARPV/ZIKV groups.

ARPV/ZIKV demonstrates exceptional safety and efficacy in immunocompetent and immunocompromised mice but very little is known about the minimal and/or optimal dose for achieving complete protection from ZIKV-induced disease. To determine the optimal ARPV/ZIKV dose that induces a protective immune response, dose de-escalation studies were performed in immune-competent mice. C57BL/6J mice were vaccinated with ARPV/ZIKV diluted in serial 1:10 dilutions ranging from 10^12^ to 10^8^ genome copies (GC; (i.e., 10^9^–10^5^ focus forming units (FFU)), or immunized with PBS, ARPV, and ZIKV PRVABC59 controls (Fig. 2a). ARPV/ZIKV-vaccinated mice administered 10^12^–10^11^ GC showed high PRNT_50_ titers as early as 1 week post immunization (Fig. 2b). At 28 days post vaccination (dpv), the ARPV/ZIKV-vaccinated 10^12^ GC group achieved a robust nAb titer of 3.41 ± 0.27 PRNT_50_, and ZIKV PRVABC59 a strong nAb titer of 3.00 ± 0.16 PRNT_50_ (Fig. 2b). Mice administered 10^12^ GC of ARPV/ZIKV did not significantly change their nAb titers post challenge when compared to PRNT_50_ at 4 weeks post immunization, while mice administered 10^11^–10^10^ GC of ARPV/ZIKV significantly increased their nAb titer post challenge (Fig. 2b). ZIKV PRVABC59 mice did not significantly change their nAb titers post challenge when compared to PRNT_50_ at 4 weeks post immunization (Fig. 2b). Our results demonstrate there was no significant difference in viremia post challenge between our healthy controls (PBS), and the ARPV/ZIKV-vaccinated 10^12^ GC and 10^11^ GC groups (Fig. 2c,d). However, there was a significant increase in viremia among the ARPV/ZIKV-vaccinated 10^9^–10^8^ GC groups at 2 dpc (Fig. 2d), and a significant increase in viremia among the ARPV/ZIKV 10^10^–10^8^ GC groups between 3 and 4 dpc, when compared to the healthy controls. There was a significant increase in ZIKV viremia at 3 and 4 dpc among the ARPV-immunized mice (Fig. 2c). Similarly, there was a significant increase in viremia at 2–4 dpc among SHAM-immunized mice (Fig. 2c). In contrast, there was no difference in weight change among the healthy controls, ARPV-immunized, ARPV/ZIKV-vaccinated 10^12^–10^8^, ZIKV PRVABC59-immunized, and SHAM-immunized groups throughout the study period (data not shown).

### Transfer of maternal antibodies from ARPV/ZIKV-vaccinated dams protects adolescent mice from ZIKV disease

**Figure 3:**
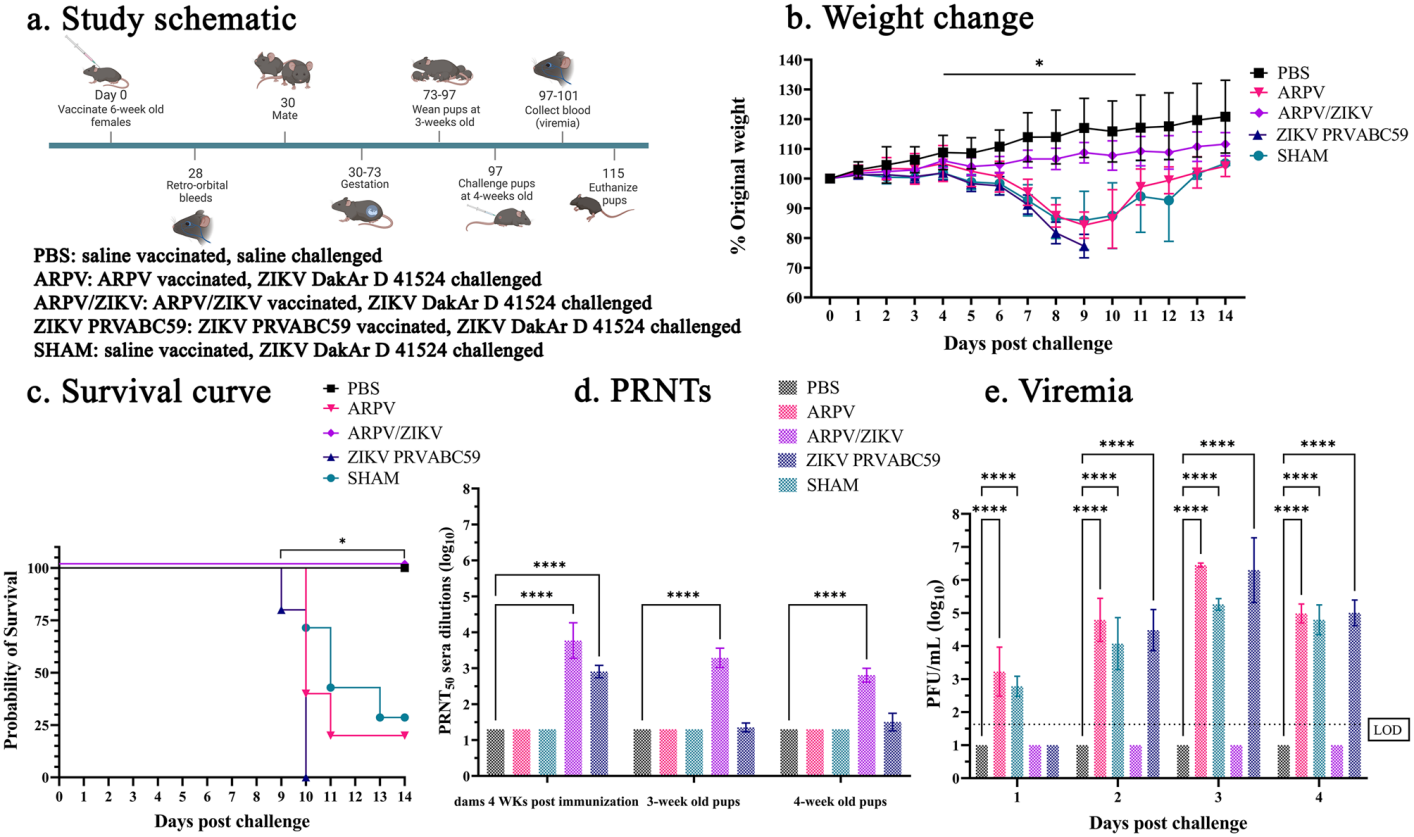
Passive transfer of maternal antibodies provides protection in 4-week-old ARPV/ZIKV-immunized mice. Six-week-old C57BL/6J mice (n = 3) were inoculated subcutaneously (s.c.) with the indicated agents or with saline (PBS), and mated with naïve males. Four-week-old pups (n = 6–10) were challenged with a lethal dose of 1 × 10^5^ plaque forming units (PFU) of ZIKV DakAr D 41524 or PBS administered s.c. at 30 days post immunization as shown in study schematic (**a**). Weight change (**b**) and survival (**c**) was measured 1–14 days post challenge. Plaque Reduction Neutralizing Tests (PRNT_50_) were done using sera collected from dams 4 weeks post immunization, and pups 3 weeks and 4 weeks post birth (**d**). Viremia was measured from sera collected on days 1–4 days post challenge (**e**). Limit of detection is indicated by the dotted line. Data points represent mean values and error bars represent the standard deviation. Significance was determined by two-way ANOVA, mixed effect analyses, or log-rank (Mantel–Cox) test when necessary. Unless otherwise marked, asterisks indicate significance compared to healthy controls (PBS): p ≤ 0.033 (*), and p ≤ 0.0001 (****). Unless otherwise marked, there was no significant difference between PBS and ARPV/ZIKV groups.

Passive transfer of antibodies to offspring may offer some protection from infectious diseases, including flavivirus infections^25–28^. We next sought to explore the passive transfer and protective efficacy of ARPV/ZIKV-induced antibodies from vaccinated dams to their offspring. To determine the rate, quantities, and protection afforded by passive transfer of maternally-derived antibodies to offspring, we immunized dams with ARPV, ARPV/ZIKV, ZIKV PRVABC59, or PBS, and challenged offspring with a lethal dose of ZIKV at ~ 4 weeks of age (Fig. 3a). Our results show that dams vaccinated with ARPV/ZIKV transferred sufficient quantities of antibodies to offer complete protection from a ZIKV challenge of their adolescent offspring. There was no significant difference in weight change, mortality, or viremia between the healthy controls (PBS) and the offspring of ARPV/ZIKV-vaccinated dams throughout the study (Fig. 3b,c,e). In stark contrast, there was a significant decrease in the weight of offspring from ZIKV PRVABC59-immunized dams between 4 and 11 days post challenge (dpc; Fig. 3b). Our results also show a significant decrease in the weight of adolescent offspring derived from ARPV-immunized dams between 7 and 11 dpc (Fig. 3b) and adolescent offspring derived from SHAM-immunized dams at 4, and 6 to 11 dpc (Fig. 3b). Significant mortality was observed among the adolescent mice from the ZIKV PRVABC59-immunized, SHAM-immunized, and ARPV-immunized dams, while the healthy controls and the offspring of ARPV/ZIKV-vaccinated dams showed 100% survival. The offspring of ARPV-immunized dams experienced 60% mortality by 9 dpc, and 80% overall mortality by 14 dpc (Fig. 3c). The offspring of ZIKV PRVABC59-immunized dams experienced 100% mortality by 10 dpc, and the offspring of naïve (SHAM-immunized) mice experienced 80% mortality by 13 dpc (Fig. 3c). These results are strongly supported by neutralizing antibody data which show adolescent mice derived from ARPV/ZIKV-vaccinated dams have high neutralizing antibody (nAb) titers of 3.23 ± 0.27 PRNT_50_ at 3 weeks after birth and persisting to 2.77 ± 0.18 PRNT_50_ at 4 weeks after birth. ARPV/ZIKV-vaccinated dams showed nAb titers of 3.76 ± 0.49 PRNT_50_ at 28 dpv, while ZIKV PRVABC59-immunized dams were significantly lower at 2.91 ± 0.17 PRNT_50_ at 28 dpv (Fig. 3d). Three-week old offspring from ARPV/ZIKV-vaccinated dams presented nAb titers of 3.23 ± 0.27 while the pups of ZIKV PRVABC59-immunized dams showed significantly lower nAb titers of 1.35 ± 0.12 PRNT_50_ (Fig. 3d). At 4 weeks, nAb titers waned in adolescent mice derived from ARPV/ZIKV-vaccinated dams to 2.77 ± 0.18 PRNT_50_ and adolescent mice derived from ZIKV PRVABC59-immunized dams waned to 1.48 ± 0.21 PRNT_50_. ZIKV PRVABC59-immunized offspring showed a significant increase in viremia when compared to healthy controls at 2 and 4 dpc. SHAM- and ARPV -immunized offspring showed a significant increase in viremia between 1 and 4 dpc (Fig. 3e).

### ARPV/ZIKV-vaccinated mice are completely protected from ZIKV-induced disease in the presence or absence of boosters

**Figure 4:**
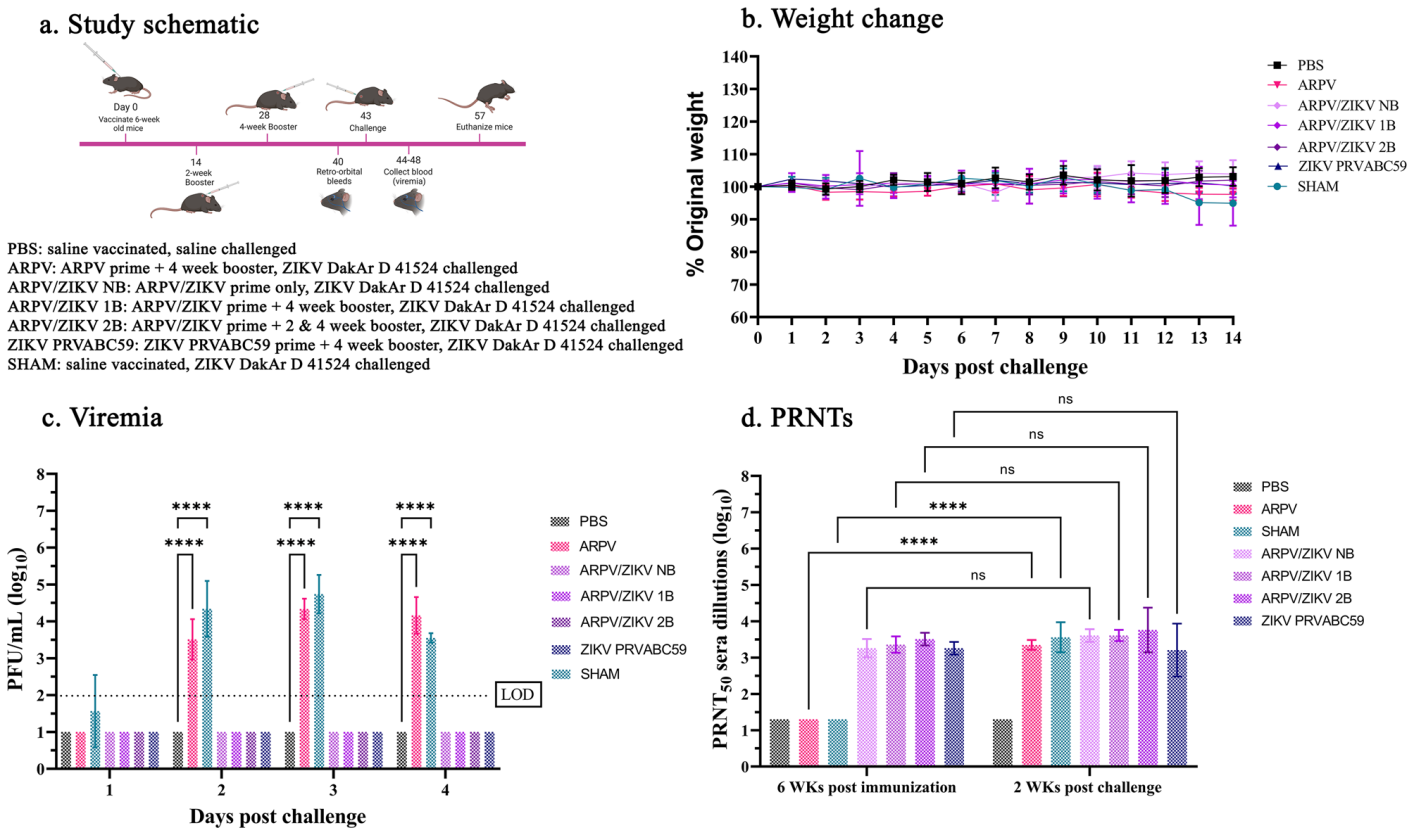
ARPV/ZIKV immunization is protective against ZIKV infection with or without boosters. Six-week-old C57BL/6J mice (n = 6) were inoculated subcutaneously (s.c.) with the indicated or with saline (PBS) as shown in the study schematic (**a**). All mice except PBS were challenged with a lethal dose of 1 × 10^5^ plaque forming units (PFU) of ZIKV DakAr D 41524 s.c. at 30 days post immunization. Weight change (**b**) and survival was measured 1–14 days post challenge. Viremia was measured from sera collected on days 1–4 post challenge (**c**). Limit of detection is indicated by the dotted line. Plaque Reduction Neutralizing Tests (PRNT_50_) were done using sera collected 6 weeks post prime and 2 weeks post challenge (**d**). Data points represent mean values and error bars represent the standard deviation. Significance was determined by two-way ANOVA and Tukey mixed-effect analyses. Unless otherwise marked, asterisks indicate significance compared to healthy controls (PBS): not significant (ns), p ≤ 0.033 (*), p ≤ 0.002 (**), p ≤ 0.0002 (***) and p ≤ 0.0001 (****). Unless otherwise marked, there was no significant difference between PBS and ARPV/ZIKV groups.

Although our studies clearly showed single-dose efficacy, sterilizing immunity was often not achieved. Herein we define sterilizing immunity as no observed statistical difference between pre- and post-challenge ZIKV nAb titers, combined with complete protection from all ZIKV-induced disease outcomes measured^29–33^. To investigate the effect of a prime-boost regimen on immunogenicity and efficacy, mice were immunized with prime only (no booster dose) (NB), or with a single booster dose administered 4-weeks post-prime (1B), or boosted both 2- and 4-weeks post-prime (2B). Mice were prime-boost immunized with PBS, ARPV and ZIKV controls as described below (Fig. 4a). Our results indicate there were no statistically significant differences in weight change among healthy control (PBS), ARPV-immunized, ZIKV PRVABC59-immunized, SHAM-immunized, and any of the ARPV/ZIKV-vaccinated booster groups throughout the study (Fig. 4b). However, there was a significant increase in viremia between 2 and 4 dpc in the ARPV- and SHAM-immunized mice (Fig. 4c). ARPV/ZIKV-vaccinated NB showed a nAb titer consistent with ZIKV PRVABC59-immunized control mice (3.26 ± 0.17 PRNT_50_). ARPV/ZIKV-vaccinated 1B mice and 2B mice produced higher nAb titers of 3.36 ± 0.23 PRNT_50_ and 3.50 ± 0.17 PRNT_50_, respectively (Fig. 4d). Post challenge, ARPV/ZIKV-vaccinated NB, 1B, and 2B groups showed PRNT_50_ nAb titers of 3.61 ± 0.16, 3.61 ± 0.16, and 3.50 ± 0.17, respectively (Fig. 4d), that were not significantly different from pre-challenge nAb titers among ARPV/ZIKV-vaccinated NB, 1B, and 2B groups, suggesting sterilizing immunity was achieved.

## Discussion

Currently, there are no FDA-approved vaccines for ZIKV despite its impact on the health of millions of people worldwide. Here we employ various in vivo and in vitro studies to explore essential characteristics of the ARPV/ZIKV vaccine candidate’s immunogenicity and efficacy in an immune-competent mouse model. Previous studies have demonstrated that chimeric ISFV-based vaccines can induce rapid and robust immune responses for various viruses^16,18,19,34,35^. Specifically, our chimeric Zika vaccine has also been shown to be highly immunogenic with evidence of a robust humoral response^21^. Our studies further confirm ARPV/ZIKV’s safety and robust immunogenicity profile and support ARPV/ZIKV as an effective vaccine candidate.

Given the global distribution of pathogenic flaviviruses, it is critically important to determine if an actively replicating VIF can influence the replication of an ISFV chimeric vaccine in vertebrate cells. Similarly, during a coinfection scenario, the presence of an ISFV may potentially affect the pathogenesis of the VIF. Recent studies show that in vivo coinfection of an ISFV and a VIF reduces pathogenesis of the VIF^20^. Our in vitro coinfection study shows ARPV/ZIKV and ARPV remain incapable of replication in vertebrate cell culture (Fig. 1a–d), even in the presence of active ZIKV replication. Although ZIKV titers increased significantly in Vero-76 cells, ARPV and ARPV/ZIKV did not increase in titer over time in either intracellular or extracellular factions (Fig. 1a–d), suggesting both viruses remain unable to replicate and egress from cells as seen with the ZIKV controls (Fig. 1a–d). Thus, ARPV/ZIKV remains a safe chimeric vaccine that can be administered without the fear of gaining replication ability, even in the presence of an ongoing ZIKV infection. Interestingly, ZIKV titers were significantly lower at 96- and 120-h post-infection (hpi) during coinfection compared to the control ZIKV infection in the intracellular but not the extracellular fraction. This suggests some degree of interference within the vertebrate cells, although it ultimately did not impact overall titers observed in the extracellular fraction. Further studies are needed to fully explore the effect of ISFVs on VIF replication and pathogenesis in vertebrate systems. Similarly, both ARPV and ARPV/ZIKV titers are reduced during coinfection with ZIKV in comparison to the control ARPV and ARPV/ZIKV infections. This also suggests some degree of interference or clearance during the active VIF replication.

Another significant advantage of the ARPV/ZIKV platform is the high viral titers achieved in cell culture at low biosafety containment, which can easily accommodate large-scale production. Establishing a minimum effective dose is essential to understanding production capacity and delivery strategy. Based on our results, the ARPV/ZIKV-vaccinated 10^12^ GC and ARPV/ZIKV-vaccinated 10^11^ GC groups showed protection from disease, in contrast to the ARPV/ZIKV-vaccinated 10^10^–10^8^ groups that showed significant viremia at 3 and 4 dpc. Thus, the minimal dose that facilitates complete protection from ZIKV-induced disease is ≥ 10^11^ GC or ≥ 10^8^ FFU per mouse. Although ARPV/ZIKV immunization with 10^12^ GC or greater offers complete protection among all outcomes measured in our studies, and showed no changes in pre- versus post-challenge nAb titers, further investigation is needed to assess for the presence of ZIKV nucleic acids in various mouse tissues and/or organs to determine if we have effectively achieved short-term sterilizing immunity. It is important to note that lower doses within the range of 10^10^–10^8^ GC should not be used to avoid the likelihood of developing sub-neutralizing Ab responses, which may lead to antibody-dependent enhancement of disease upon natural infection.

We also sought to determine rates of passive antibody transfer from dams to offspring, compare neonatal and maternal antibody levels, and evaluate the degree of protection this passive immunity affords to offspring. Adolescent mice born to dams vaccinated with ARPV/ZIKV presented with high nAb titers and remained healthy after ZIKV challenge. Passive transfer of nAbs was documented in 100% of the offspring of ARPV/ZIKV-vaccinated and ZIKV PRVABC59-immunized mice (n = 10). As expected, levels of nAb titers estimated in offspring correlated well with levels estimated in their respective dams. Antibody titers rapidly waned after weaning, ultimately leading to the absence of any protection in the offspring of ZIKV PRVABC59-immunized dams. Further studies are needed to determine the longevity of protection afforded by passive transfer of maternal antibodies after ARPV/ZIKV immunization. Considering mammals, including humans, can confer passive transfer of maternal antibodies^22^, it would be beneficial to determine whether ARPV/ZIKV vaccination of mothers and subsequent milk-derived antibodies have any significant impact on protecting newborns from ZIKV infection after birth in non-human primate models and mice both long-term and short-term.

Finally, our booster study revealed that ARPV/ZIKV boosters did not significantly increase nAb titers when measured 6 weeks post-prime vaccination. This data suggests boosters have no impact on short-term immunogenicity. Our data also shows that nAb titers in all ARPV/ZIKV-vaccinated groups did not significantly increase after ZIKV challenge, indicating the presence of sterilizing immunity in these groups, supporting the results of our dose de-escalation studies above. These data suggest that ARPV/ZIKV immunization generates sufficient immunogenicity to completely prevent ZIKV infection, such that the challenge has no impact on circulating ZIKV-specific nAb responses. ZIKV PRVABC59-immunized mice were also boosted at 4 weeks post-prime and showed comparable nAb titers to the ARPV/ZIKV-vaccinated NB and 1B groups. ARPV-immunized mice were also boosted at 4 weeks post-prime and showed a significant increase in ZIKV-specific nAb titers post challenge (Fig. 4d). As observed in our earlier studies, all ARPV/ZIKV-vaccinated and ZIKV PRVABC59-immunized mice were completely protected from any ZIKV-induced disease after challenge^21^. Further studies are nonetheless needed to determine the impact of boosters on long-term immunity and durability of protection.

There are several ZIKV vaccines in clinical trials^11,12,36–39^ including nucleic acid, inactivated, and live-attenuated vaccines. While these platforms offer several advantages, they also present some challenges such as sub-optimal immunogenicity, potential reversion to virulence, requiring boosters, and achieving low production titers^37,40^. ARPV/ZIKV does not present these obstacles and can easily be produced in a low-containment facility in cell culture. Research is still needed to determine the optimal strategy for storing, transporting, formulating, and administering this vaccine. One drawback of this platform is that ARPV/ZIKV is currently produced in *Aedes albopictus* cells (C6/36), which is not an FDA-approved cell substrate. Future work is needed to adapt ARPV/ZIKV to an approved cell substrate, such as Sf9 cells (*Spodoptera frugiperda*, the fall armyworm), or seek regulatory approval for C6/36-based production^41–44^.

In summary, optimizing production capacity and efficacy of ISFV-based vaccine candidates is critically important for their successful licensure, approval, and dissemination to the public. This article highlights that with the use of our vaccine candidate, a dose of 10^12^ GC or 10^9^ FFU will offer complete protection from ZIKV-induced disease in murine models, including boosters does not significantly impact short-term immunity, and maternal antibodies are effectively transferred to newborns in protective quantities. The coinfection studies reported here also show that ARPV/ZIKV remains a very safe vaccine. Zika, like many other tropical arboviral diseases, impacts developing countries that do not have funding or the capability to eradicate diseases using conventional approaches. This vaccine platform aims to offer a cheap and stable alternative that can be grown at low containment without the need for boosters.

## Methods

### Cell lines and viruses

*Aedes albopictus* (C6/36) and African green monkey kidney cells (Vero-76) were purchased from ATCC (Manassas, VA, USA) and maintained according to ATCC guidelines. C6/36 cells were maintained in Dulbecco’s Modified Eagle’s Medium (DMEM, Corning, Corning, NY) with 10% fetal bovine serum (FBS), 100 U/mL of penicillin and 100 μg/mL of streptomycin, 1% tryptose phosphate broth, and 1X concentration of non-essential amino acids, at 28 °C with 5% CO_2_. Vero-76 cells were maintained in Dulbecco’s Modified Eagle’s Medium (DMEM, Corning, Corning, NY) with 5% fetal bovine serum (FBS), 100 U/mL of penicillin and 100 μg/mL of streptomycin, at 37 °C with 5% CO_2_. Zika virus strains PRVABC59 and DakAr D 41524 were obtained from Dr. Nisha Duggal (Virginia Tech, Blacksburg, VA, USA). ARPV was originally isolated from *Psorophora albipes* mosquitoes collected from the Aripo savannahs on the Caribbean island of Trinidad^20^. The ARPV/ZIKV chimera was constructed as described previously^21^.

### Viral replication kinetics and quantification

Vero-76 cells were infected in triplicate with the following combinations of viruses: ZIKV DakAr D 41,524 (4.0 × 10^6^ GC) & ARPV(3.4 × 10^6^ GC), ZIKV DakAr D 41524 (8.7 × 10^5^ GC) and AZ (6.5 × 10^6^ GC) for coinfected groups, ZIKV DakAr D 41524 (2.4 × 10^6^ GC), ARPV (5.7 × 10^6^ GC), and AZ (5.4 × 10^6^ GC) for control groups. Cells were infected at a target multiplicity of infection (MOI) of 0.1, and intracellular and supernatant fractions were taken at 0, 3, 6, 12, 24, 36, 48, 72, 96, and 120 h post-infection (hpi). Extracellular samples were taken from supernatant and intracellular samples were extracted by applying lysis buffer directly to washed cells for ten minutes following removal of supernatant. RNA was isolated from each sample and viral replication kinetics were assessed in both intracellular and extracellular fractions as previously described^20^. RNA extractions were performed using QIAmp Viral RNA Mini kits (QIAGEN) according to the manufacturer’s instructions. RT-qPCR was performed in triplicate using iTaq™ Universal Probes One-Step kit (Bio-Rad Laboratories, Hercules, CA, USA) according to the manufacturer’s guidelines. The ARPV and ZIKV primers and probes used were previously described^21^. Growth kinetics for each virus are presented as log_10_ fold change over time, where the fold change was estimated by dividing the virus titer at the indicated timepoint by the titer measured at the 0 h time point for that respective virus.

### Virus and antibody quantification

Plaque assays and plaque reduction neutralization tests (PRNT) were performed as previously described^45^ to quantify infectious virus particles and neutralizing antibodies. PRNT assays were performed using ZIKV strain PRVABC59. Control groups include PBS (saline vaccinated and saline challenged), ARPV-immunized (ARPV immunized and ZIKV DakAr challenged), SHAM-immunized (PBS immunized and ZIKV DakAr challenged), ARPV/ZIKV (ARPV/ZIKV vaccinated and ZIKV DakAr challenged), and ZIKV PRVABC59 (ZIKV PRVABC59 immunized and ZIKV DakAr challenged). ZIKV PRVABC59 is a mouse attenuated strain from Dr. Nisha Duggal as previously described^21^. Mice were bled retro-orbitally 1 week, 4 weeks, or 6 weeks post prime immunization as needed and 2 weeks post challenge for PRNTs. ARPV/ZIKV was also quantified using a focus forming assay (FFA) with 4G2 antibodies. The FFA was performed as previously described^46^.

### Assessment of optimal doses

Four-week-old C57BL/6J mice were divided into nine groups (n = 6/group; Jackson Laboratory, Bar Harbor, ME, USA) and inoculated s.c. with PBS, 10^9^ GC of ARPV, 10^7^ GC of ZIKV PRVABC59, or dose-descalation was carried out at doses ranging from 10^12^ to 10^8^ GC (i.e., 10^9^–10^5^ FFU) of ARPV/ZIKV. Control groups include PBS (saline vaccinated and saline challenged), ARPV-immunized (ARPV immunized and ZIKV DakAr challenged), SHAM-immunized (PBS immunized and ZIKV DakAr challenged), ARPV/ZIKV (ARPV/ZIKV vaccinated and ZIKV DakAr challenged), and ZIKV PRVABC59 (ZIKV PRVABC59 immunized and ZIKV DakAr challenged). Mice were bled weekly post immunization and 2 weeks post challenge and serum stored for ZIKV-specific PRNTs. nAb titers measured at 2 weeks post challenge were used to assess for sterilizing immunity, and nAb titers estimated pre-challenge were used to correlate protection efficacy. MAR1-5A3 was administered as described below. Weight loss, disease progression, and survival were assessed for 14 dpc.

### Passive transfer of antibodies from dams to offspring

Six-week-old female C57BL/6J mice (strain #000664) were purchased from Jackson Laboratory (Bar Harbor, ME, USA) and inoculated subcutaneously (s.c.) with either 10^8^ GC of ARPV (n = 3), 10^7^ GC of ZIKV PRVABC59 (n = 5), 10^12^ GC (10^9^ FFU) of ARPV/ZIKV (n = 4), or an equivalent volume of phosphate-buffered saline (PBS) (n = 3; healthy controls). Control groups include PBS (saline vaccinated and saline challenged), ARPV-immunized (ARPV immunized and ZIKV DakAr challenged), SHAM-immunized (PBS immunized and ZIKV DakAr challenged), ARPV/ZIKV (ARPV/ZIKV vaccinated and ZIKV DakAr challenged), and ZIKV PRVABC59 (ZIKV PRVABC59 immunized and ZIKV DakAr challenged). ZIKV PRVABC59 is a mouse-attenuated strain from Dr. Nisha Duggal as previously described^18^.

Mice were bled retro-orbitally 28 dpv and serum stored for ZIKV-specific plaque reduction neutralization tests (PRNTs). At 30 dpv dams were mated with C57BL/6J males and monitored for signs of pregnancy and birth for 3 weeks. A subset number of pups were sacrificed at 3 weeks of age to collect sera for PRNTs when available. Pups were weaned and bled at 21 days of age and again at 4 weeks of age immediately preceding ZIKV challenge. A MAR1-5A3 anti-mouse IFNRαβ^-/-^ blocking antibody (Leinco Technologies; St. Louis, MO, USA) was administered intraperitoneally (i.p.) to adolescent mice at doses and intervals previously described^24^. Mice were challenged s.c. at approximately 4 weeks of age with 10^5^ plaque forming units (PFU) of ZIKV strain DakAr D 41524. Blood was collected daily from each group 1–4 days post challenge (dpc) to quantify viremia (Fig. 2a). Weight change, disease progression, and survival were measured for 14 dpc.

### Assessment of booster doses

Four-week-old C57BL/6 J mice were purchased from Jackson Laboratory and divided into seven groups (n = 6) before s.c. inoculation. Control groups were inoculated (s.c.) with 10^9^ GC of ARPV, 10^7^ GC of ZIKV PRVABC59, or PBS. ARPV/ZIKV groups received a dose of 10^11^ GC (10^8^ FFU) of ARPV/ZIKV and groups include: mice administered a prime immunization only (no booster dose) (NB), mice with a single a single booster dose administered 4-weeks post-prime immunization (1B), and mice boosted both 2- and 4-weeks post-prime (2B). ZIKV PRVABC59 is a mouse attenuated strain from Dr. Nisha Duggal as previously described^21^. ZIKV PRVABC59 and ARPV mice were boosted at 4 weeks post-prime. Mice were bled weekly to measure ZIKV-specific neutralizing antibody titers. Mice received MAR1-5A3 as described above then challenged s.c. with 10^5^ PFU of Zika DakAr D 41524 or PBS 2 weeks after the last booster (i.e., 6 weeks post prime immunization). Retro-orbital bleeds were performed daily for 4 dpc to assess viremia (Fig. 4a). Weight change, disease progression, and survival were assessed for 14 dpc as described above.

### Statistical analysis

Data normality was assessed using a combination of Q–Q plot, and box-plot analyses. Data were normalized by log10 transformation when necessary. One-way and two-way ANOVAs, and mixed-effects analyses were performed to assess significance along with multiple comparisons (α = 0.05). Kaplan–Meier survival curves were analyzed by a log-rank Mantel–Cox test. Statistical analyses were performed in GraphPad Prism 9.3.

### Ethical approval and informed consent

All experimental protocols were approved by the Virginia Tech Institutional Biosafety Committee. All animal study protocols and experiments were approved by Virginia Tech’s Institutional Animal Care and Use Committee (IACUC). All animal experiments were performed in compliance with the guidelines of the Virginia Tech’s IACUC. All animal experiments were performed in accordance to ARRIVE guidelines.

## Data Availability

All reagents, data and associated protocols are available from the corresponding author upon request.
